# Draft genomes of two *Pseudomonas* spp. strains isolated from tomato plants

**DOI:** 10.1128/mra.01037-25

**Published:** 2026-02-27

**Authors:** Darío Alvarado-Rodríguez, Gabriel Vargas Asensio, Mónica Blanco-Meneses

**Affiliations:** 1 Crop Protection Research Center (CIPROC), Faculty of Agri-Food Sciences, University of Costa Rica 27915, San José, Costa Rica; University of Strathclyde, Glasgow, United Kingdom

**Keywords:** plant pathogenic bacteria, *Pseudomonas alliivorans*, *Pseudomonas capsici*

## Abstract

We present the draft genome sequences of *Pseudomonas alliivorans* LTM 13.1.2 (6.1 Mb) and *Pseudomonas capsici* LTM 78.3.2 (5.8 Mb) isolated from tomato plants in Costa Rica. These draft genomes provide important insights into the virulence factors and antibiotic resistance genes of these plant pathogens.

## ANNOUNCEMENT

Bacteria from the genus *Pseudomonas* are known pathogens of tomato and other crops. *Pseudomonas alliivorans* LTM 13.1.2 and *Pseudomonas capsici* LTM 78.3.2 were isolated from necrotic stem and leaf lesions of diseased tomato plants collected in 2021 and 2023 in Paraiso (Cartago) and Santa Bárbara (Heredia), Costa Rica. Plant samples were surface sterilized with 1% sodium hypochlorite (30 s), 70% ethanol (1 min), and rinsed three times with sterile distilled water. Tissues from lesion margins were aseptically excised, macerated in sterile 0.85% saline solution, and streaked onto nutrient agar (NA; OXOID, CM0003; Thermo Scientific). Plates were incubated at 28°C for 24–72 h, and single colonies were subcultured on fresh NA to obtain pure isolates. Isolates were stored in nutrient broth (OXOID, CM0001; Thermo Scientific) with 35% glycerol at −80°C and reactivated on NA plates at 28°C for 24 h before DNA extraction.

Genomic DNA was extracted from single colonies using a modified cetyltrimethylammonium bromide (CTAB) method (1, 2). Colonies were suspended in 150 µL of extraction buffer (0.35 M sorbitol, 0.1 M Tris-HCl pH 7.5, 0.005 M EDTA pH 8, 0.02 M sodium bisulfite) and 150 µL of lysis buffer (2 M NaCl, 0.2 M Tris-HCl pH 7.5, 0.05 M EDTA pH 8, 2% CTAB), vortexed for 15 s, and incubated at 55°C for 60 min. DNA was purified twice with chloroform:isoamyl alcohol (24:1), precipitated with cold 95% ethanol and 3 M sodium acetate at −20°C overnight, centrifuged at 12,000 rpm for 20 min, washed with 70% ethanol, air-dried, resuspended in 50 µL Tris-EDTA (TE) buffer (10 mM Tris-HCl, 1 mM EDTA, pH 8.0), and stored at −20°C. DNA concentration and purity were assessed using a NanoDrop One spectrophotometer (Thermo Scientific).

DNA libraries were prepared using the Illumina DNA Prep Kit, and genome sequencing was performed using the Illumina NovaSeq Plus X (2 × 151) paired-end method at SeqCenter (Pittsburgh, Pennsylvania, USA). Raw reads were trimmed with Trimmomatic v0.36 (3) using *ILLUMINACLIP:TruSeq3-PE-2.fa:4:30:10 MINLEN:30* and assembled with Unicycler v0.4.8 (4) using *--min_fasta_length 1000*. The *Pseudomonas alliivorans* LTM 13.1.2 genome (6.1 Mb) had 143× coverage, and the *P. capsici* LTM 78.3.2 genome (5.8Mb) had 146× coverage. Genome quality was assessed with QUAST v5.2.1 (5). Completeness and contamination were assessed with Checkm2 v1.0.1 (6) using the uniref100.KO.1 database. Taxonomic classification was conducted using GTDB-tk v2.0.0 (7) based on average nucleotide identity (ANI) and functional annotation with EggNOGmapper v2.1.12 (8). Additional genomic features, such as secondary metabolites, virulence factors, and antibiotic resistance, were annotated using antiSMASH v8.0 (9) in strict detection mode, MetaVF toolkit (10), and the resistant gene identifier RGI v6.1.3 tools implemented by the Comprehensive Antibiotic Resistance Database (CARD) (11), respectively. Default parameters were used, except where otherwise noted.

The *P. alliivorans* LTM 13.1.2 genome shows an ANI of 98.86% when compared to *P. alliivorans* 20GA0068^T^ (GCA_017826695.1) and contains 28 candidate virulence factors predicted to be involved in colonization, immune evasion, and housekeeping functions, 11 predicted secondary metabolite gene clusters, and six predicted antibiotic resistance genes associated with target alteration, efflux, and inactivation. The *P. capsici* LTM 78.3.2 genome has an ANI of 99.53% relative to *P. capsici* Pc19-1^T^ (GCF_017165765.1) and contains 30 candidate virulence factors, 14 predicted secondary metabolite gene clusters, and four predicted antibiotic resistance genes, all related to efflux mechanisms. Genome statistics and features are summarized in Table 1.

**TABLE 1:** Genome statistics and features of *Pseudomonas alliivorans* LTM 13.1.2 and *Pseudomonas capsici* LTM 78.3.2

	*P. alliivorans* LTM 13.1.2	*P. capsici* LTM 78.3.2
Total assembly length (bp)	6,158,523	5,872,844
No. of raw paired-end reads	5,869,840	5,721,138
No. of contigs	52	54
Largest contig	674,345	506,609
GC%	59.1	58.37
N50	249,463	182,980
N75	166,834	154,069
L50	8	9
L75	15	18
Predicted genes	5,729	5,188
Completeness	99.98	100
Contamination	1.26	0.15
Genes annotated in KEGG^*a*^	3,194	3,044
Genes annotated in CAZy^*b*^	57	44
Genes annotated in PFAMs^*c*^	4,810	4,441
BGCs^*d*^	11	14
GOs^*e*^	1,238	1,193
Virulence factors	28	30
Antibiotic resistance genes	6	4
COG categories^*f*^	21	22

^*a*^ KEGG, Kyoto Encyclopedia of Genes and Genomes.

^*b*^ CAZy, Carbohydrate-Active Enzymes database.

^*c*^ PFAMs, Protein Families database.

^*d*^ BGCs, biosynthetic gene clusters.

^*e*^ GOs, Gene Ontology terms.

^*f*^ COG categories, functional categories of Clusters of Orthologous Groups.

## Data Availability

Data can be found in the NCBI BioProject accession number PRJNA1314254. This Whole Genome Shotgun project has been deposited at GenBank under accession numbers JBQWBA000000000 (*P. capsici* LTM 78.3.2) and JBQWBB000000000 (*P. alliivorans* LTM 13.1.2). The version described in this paper is the first version. The raw reads are available under SRA accession numbers SRS26434160 (P. alliivorans LTM 13.1.2) and SRS26434161 (*P. capsici* LTM 78.3.2). The described genome annotations are available at Zenodo (DOI: https://doi.org/10.5281/zenodo.17547179).
